# A national snapshot of the impact of clinical depression on post-surgical pain and adverse outcomes after anterior cervical discectomy and fusion for cervical myelopathy and radiculopathy: 10-year results from the US Nationwide Inpatient Sample

**DOI:** 10.1371/journal.pone.0258517

**Published:** 2021-10-15

**Authors:** Jiang Chen, Jin-Yu Li, Gui-Hua Tian, Rui-Jin Qiu, Xue-Qian Zhao, Xue-Shi Di, Qiao-Mei Yuan, Shui-Wen Long, Yu Ran, Yu-Song Jia, Hong-Cai Shang

**Affiliations:** 1 Hunan University of Chinese Medicine, Changsha, Hunan, China; 2 Department of Orthopaedics, Dongzhimen Hospital, Beijing University of Chinese Medicine, Beijing, China; 3 Key Laboratory of Chinese Internal Medicine of Ministry of Education and Beijing, Dongzhimen Hospital, Beijing University of Chinese Medicine, Beijing, China; University of California San Francisco, UNITED STATES

## Abstract

Depression is associated with poorer outcomes in a wide spectrum of surgeries but the specific effects of depression in patients undergoing cervical spine surgery are unknown. This study aimed to evaluate the prevalence and impact of pre-surgical clinical depression on pain and other outcomes after surgery for cervical degenerative disc disease using a national representative database. Data of patients with cervical myelopathy and radiculopathy were extracted from the 2005–2014 US Nationwide Inpatient Sample (NIS) database. Included patients underwent anterior discectomy and fusion (ACDF). Acute or chronic post-surgical pain, postoperative complications, unfavorable discharge, length of stay (LOS) and hospital costs were evaluated. Totally 215,684 patients were included. Pre-surgical depression was found in 29,889 (13.86%) patients, with a prevalence nearly doubled during 2005–2014 in the US. Depression was independently associated with acute or chronic post-surgical pain (aOR: 1.432), unfavorable discharge (aOR: 1.311), prolonged LOS (aOR: 1.152), any complication (aOR: 1.232), respiratory complications/pneumonia (aOR: 1.153), dysphagia (aOR: 1.105), bleeding (aOR: 1.085), infection/sepsis (aOR: 1.529), and higher hospital costs (beta: 1080.640) compared to non-depression. No significant risk of delirium or venous thrombotic events was observed in patients with depression as compared to non-depression. Among patients receiving primary surgery, depression was independently associated with prolonged LOS (aOR: 1.150), any complication (aOR:1.233) and postoperative pain (aOR:1.927). In revision surgery, no significant associations were found for prolonged LOS, any complication or pain. In conclusion, in the US patients undergoing ACDF, pre-surgical clinical depression predicts post-surgical acute or chronic pain, a slightly prolonged LOS and the presence of any complication. Awareness of these associations may help clinicians stratify risk preoperatively and optimize patient care.

## Introduction

Depression and anxiety are the two most commonly diagnosed mental health conditions worldwide, often manifesting together. In 2012, the World Health Organization (WHO) ranked depression as the third major contributor to the global disease burden and approximated that by 2030 it would rank first [1]. In the United States, the prevalence of depression increased significantly between 2005 and 2015, with contributing factors varying between different population subgroups [2].

Recent studies have demonstrated that depression is associated with increased complications following various orthopedic procedures. For hip and knee arthroplasty, depression was an independent risk factor for postoperative pain, adverse events and non-routine discharge [3–5]. For total shoulder arthroplasty, clinical depression diagnosed prior to surgery independently predicted postoperative delirium, anemia and infection [6]. Fusions performed for lumbar degenerative disease in patients with comorbid psychiatric conditions had less favorable short-term outcomes (e.g., unfavorable discharge, neurologic and vascular complications and renal failure) than in those without concomitant psychiatric disease [7]. Results of a prior review revealed that several specific interacting factors (i.e., pain, disability, mental health status, lack of information and return to work) were associated with depression and anxiety symptoms both before and after spinal surgery, although having more information about their condition seemed to help regulate patients’ depression and anxiety to some extent [8]. It was reported surgery for spinal stenosis relieved depressive symptoms in some patients, but poorer clinical outcomes were common in patients with more persistent postoperative depression [9].

In previous studies, the focus has been on primarily joint arthroplasty and lumbar spine surgery, while less attention has been given to effects of concomitant depression on outcomes of degenerative cervical spine surgery, which is being performed increasingly [10]. For treatment of symptomatic cervical myelopathy and radiculopathy, anterior cervical discectomy and fusion (ACDF), posterior cervical decompression and fusion (PCF), or a combination of the two (front-back) are performed most often [10, 11]. A combination of different mental health conditions was linked to greater risk of readmission and revision in cervical spine surgical patients in a previous report [12], and inconsistent results were shown between other studies examining the effects of preoperative psychiatric disorders on surgical outcomes of cervical spine surgery [13–16], Limitations of these studies included either small sample size, limited outcomes evaluated or lack of differentiation between depression and other psychiatric conditions. Importantly, no comprehensive investigation of clinical depression in degenerative cervical spine surgery has yet been performed in a nationwide population. Therefore, this study was designed and conducted to evaluate the national prevalence and impact of depression relative to surgeries performed to treat degenerative cervical myelopathy and radiculopathy in the United States.

## Methods

### Study design and data source

This population-based, retrospective observational study extracted all data from the Nationwide Inpatient Sample (NIS) database. As the largest all-payer, continuous inpatient care database in the United States, the NIS comprises about 8 million hospital stays each year drawn from about 1050 hospitals in 44 US states, which represents a 20% stratified sample of US community hospitals using the American Hospital Association definition. Sponsorship is provided by the Agency for Healthcare Research and Quality (AHRQ) and the program is administered by the Healthcare Cost and Utilization Project (HCUP) of the National Institutes of Health (NIH) [17], Data provided for all patients include demographics, primary and secondary diagnoses, primary and secondary procedures, admission and discharge status, expected payment source, duration of hospital stay (length of stay [LOS]), and hospital-related characteristics (i.e., bed size/location/teaching status/hospital region). All patients identified as having cervical degenerative disease were initially considered for inclusion in the analytic sample.

### Ethics statement

All data were obtained through request to the online Healthcare Cost and Utilization Project (HCUP) Central Distributor (available at: https://www.distributor.hcup-us.ahrq.gov/). This study conforms to the data-use agreement between NIS and HCUP. Analysis of NIS data uses completely deidentified data without risk of loss of confidentiality. An initial expedited review by the Institutional Review Board of the Dongzhimen Hospital, Beijing University of Chinese Medicine deemed that the deidentified data were exempt from further ethical review, and waived the requirement for informed consent.

### Study population

Adults ≥18 years old admitted to U.S. hospitals between 2005 and 2014 with a primary diagnosis of cervical degenerative diseases, including cervical myelopathy and/or radiculopathy, were identified in the NIS database using the International Classification of Diseases, 9th Revision (ICD-9) diagnostic codes. Patients undergoing anterior discectomy and fusion (ACDF) were further identified using previously described primary procedure codes [18], Only patients who had been admitted electively were included. Patients with 1) metastatic or nonmetastatic cancer, lymphoma, or leukemia, 2) schizophrenia and bipolar disorders, 3) any vertebral fractures, 4) trauma, 5) paralysis, 6) multi-level fusion, 7) emergent admission, and 8) codes for posterior procedures were excluded, as well as patients without complete data for main outcomes or all variables of interest. For analysis, the included patients were further categorized into depressive and non-depressive groups, according to relevant ICD-9 codes in the medical records. Details of relevant codes are shown in S1 Table.

### Main outcome measures and variables

The main study endpoints were: 1) unfavorable discharge (defined as discharge to nursing facility, extended care facility, or hospice); 2) prolonged length of stay (defined as LOS > = 3 days); 3) presence of any or specific postoperative complications; and 4) hospital costs.

Postoperative complications were identified using ICD-9 and Clinical Classifications Software (CCS) codes [19], including: postoperative acute or chronic pain, delirium, dysphagia, cardiovascular complications, venous thromboembolic events, respiratory complications and pneumonia, acute kidney injury, digestive system complications, bleeding complications and infection/sepsis. Details of the relevant codes are shown in S1 Table.

#### Covariates

Patients’ characteristics included age, gender, household income level, race/ethnicity, insurance status (primary payer), diagnosis (myelopathy, radiculopathy and other degenerative conditions), procedure type, and comorbidities. Comorbidities were identified according to AHRQ comorbidity measures listed in the database, which were also determined using ICD-9 diagnostic codes with algorithms validated by Elixhauser [20], excluding items already included as patients’ characteristics. In addition to AHRQ comorbidities, opioid dependence/abuse, other drug dependence/abuse, obstructive sleep apnea, osteoporosis, tobacco use and anxiety were also included as covariates (S1 Table). Data of hospital-related characteristics (i.e., bed size, location/teaching status, hospital region) and the annual caseload for cervical spine surgery were also extracted from the database as part of the NIS comprehensive patient data.

### Statistical analysis

All categorical variables are expressed as counts (percentages). Continuous variables are presented as means with standard deviation (SD) and analyzed using Student’s t-test. Comparisons of proportions between groups for categorical variables were performed using Pearson’s chi-square test or Fisher’s exact test, with missing values excluded. Univariate and multivariate analysis were performed to determine associations between study variables and occurrence of 1) unfavorable discharge, 2) prolonged length of stay, 3) any or specific postoperative complications and 4) hospital costs. Additional stratified analyses were conducted according to procedure type. SAS statistical software version 9.4 (SAS, Cary, NC, USA) was used for all statistical analyses. The results are presented as odds ratios (ORs) and 95% confidence intervals (CIs). Two-sided *p*-values less than 0.05 were established as statistical significance.

## Results

### Study population

**Fig 1:**
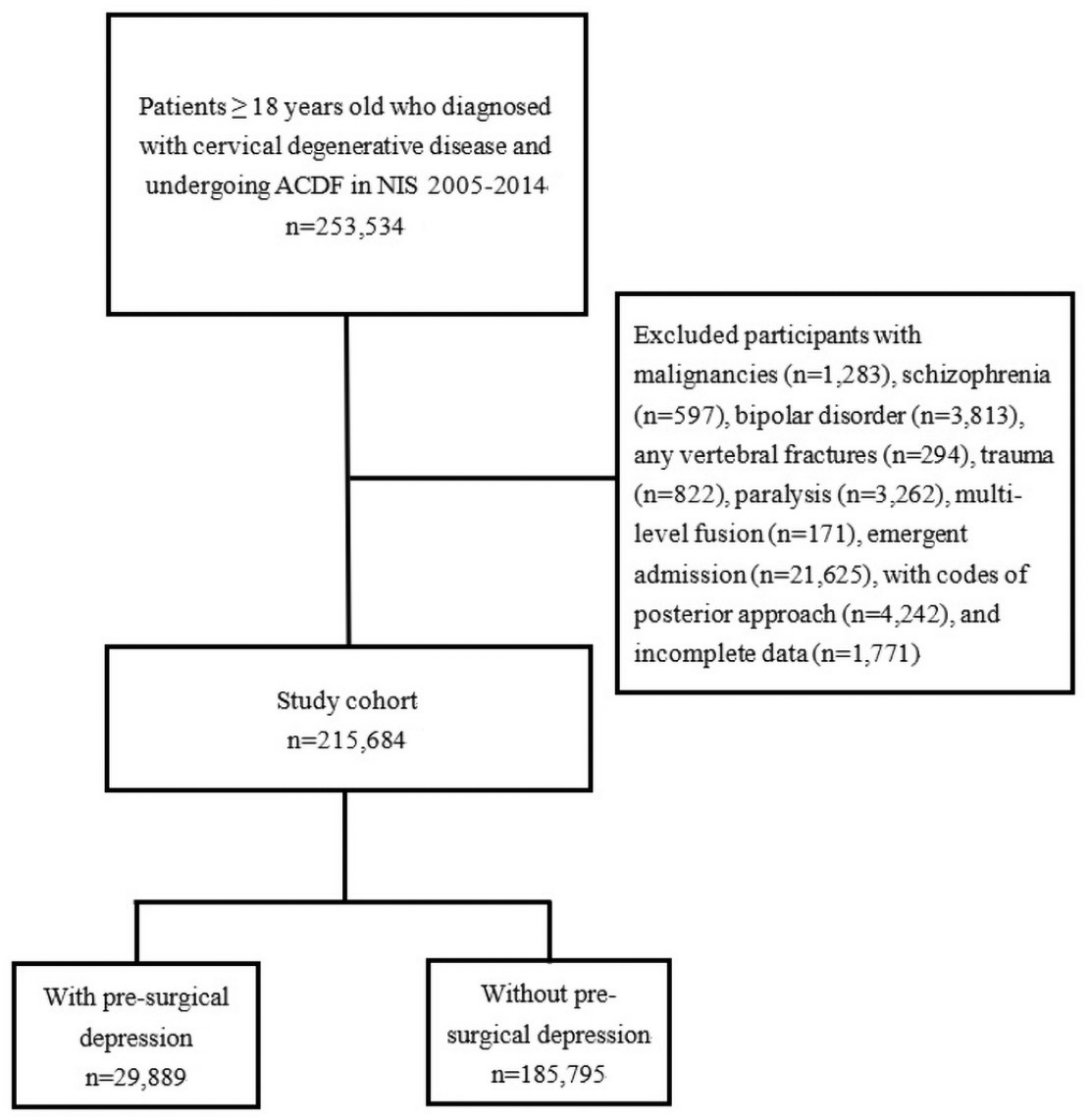
Flow diagram of study selection process.

The process of study selection is summarized in Fig 1. Initial screening of the NIS database from 2005 to 2014 identified total of 77,394,845 eligible patients. Among these, 253,534 were adults 18 years old or older diagnosed with cervical degenerative diseases and admitted for surgical treatment by ACDF. After excluding patients with malignancies, schizophrenia and bipolar disorder, any vertebral fractures, trauma, paralysis, multi-level fusions, emergent admissions, posterior procedures and incomplete data for main outcomes and variables, a total of 215,684 hospitalized patients remained as the primary cohort whose data comprised the analytic sample (Fig 1).

### Demographic and clinical characteristics

**Fig 2:**
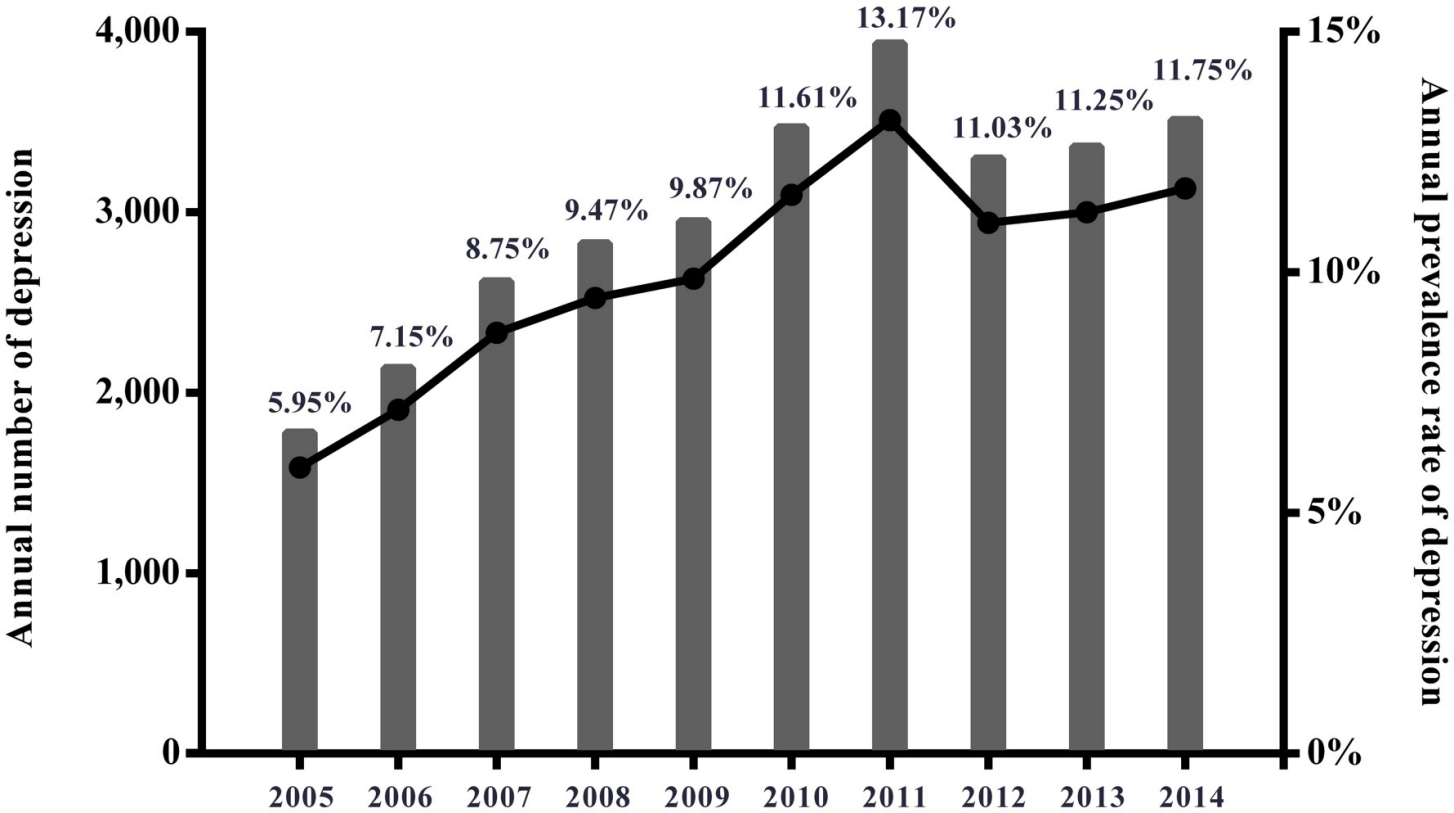
Prevalence of depression in patients undergoing ACDF by year.

Patients’ baseline demographic, clinical and hospital-related characteristics are summarized in Table 1. Pre-surgical clinical depression was noted in 29,889 (13.86%) patients. The majority of patients were aged 41–54 years (46.21%), female (69.02%), White (87.48%), with income at the second quartile (28.15%), covered by private insurance (552.05%), and received primary procedure (98.22%). Mean patient age was 53.04±10.59 and 53.31±11.73 in the depression and non-depression groups, respectively (P<0.001). Other significant differences observed between patients with and without depression were gender, income, race/ethnicity, insurance status, diagnosis of degenerative conditions, procedure types, number of comorbidities and hospital-related characteristics (all P < 0.001). The depression group had greater proportions of all major comorbidities than did the non-depression group (Table 1). Prevalence of depression increased from 5.95% in 2005 to 11.75% in 2014 among patients undergoing ACDF (Fig 2).

**Table 1:** Characteristics of 215864 patients undergoing ACDF in the NIS database 2005–2014.

Characteristics	Total (n = 215684)	Depression		P-value
		No (n = 185795)	Yes (n = 29889)	
**Age**	53.27±11.58	53.31±11.73	53.04±10.59	**<0.0001**
18–40	27618 (12.80%)	24248 (13.05%)	3370 (11.28%)	**<0.0001**
41–54	95767 (44.40%)	81956 (44.11%)	13811 (46.21%)	
55–64	52612 (24.39%)	44470 (23.93%)	8142 (27.24%)	
65–76	30524 (14.15%)	26753 (14.40%)	3771 (12.62%)	
75+	9163 (4.25%)	8368 (4.50%)	795 (2.66%)	
**Gender**				**<0.0001**
Female	114686 (53.18%)	94059 (50.63%)	20627 (69.02%)	
Male	100967 (46.82%)	91707 (49.37%)	9260 (30.98%)	
**Income**				**<0.0001**
Lowest quartile	49071 (23.25%)	42044 (23.13%)	7027 (23.96%)	
Second quartile	56075 (26.57%)	47818 (26.31%)	8257 (28.15%)	
Third quartile	56113 (26.58%)	48369 (26.61%)	7744 (26.40%)	
Fourth quartile	46817 (23.60%)	43516 (23.94%)	6301 (21.48%)	
**Race/ethnicity**				**<0.0001**
White	147814 (82.88%)	126209 (82.14%)	21605 (87.48%)	
Black	15041 (8.43%)	13624 (8.87%)	1417 (5.74%)	
Hispanic	8420 (4.72%)	7434 (4.84%)	986 (3.99%)	
Asian	1915 (1.07%)	1792 (1.17%)	123 (0.50%)	
Others (Native America + others)	5153 (2.89%)	4586 (2.98%)	567 (2.30%)	
**Insurance status / Primary Payer**				**<0.0001**
Medicare	53932 (25.07%)	45143 (24.36%)	8789 (29.47%)	
Medicaid	11939 (5.55%)	9368 (5.05%)	2571 (8.62%)	
Private	123372 (57.34%)	107851 (58.20%)	15521 (52.05%)	
Self/ no charge /others	25903 (12.04%)	22964 (12.39%)	2939 (9.86%)	
**Diagnosis**				**<0.0001**
Myelopathy	119860 (55.57%)	104230 (56.10%)	15630 (52.29%)	
Radiculopathy	66035 (30.62%)	56464 (30.39%)	9571 (32.02%)	
Other degenerative condition	29789 (13.81%)	25101 (13.51%)	4688 (15.68%)	
**Procedure**				**<0.0001**
Primary	212822 (98.67%)	183464 (98.75%)	29358 (98.22%)	
Revision	2862 (1.33%)	2331 (1.25%)	531 (1.78%)	
**Comorbidities**				
Alcohol abuse	1936 (0.90%)	1466 (0.79%)	470 (1.57%)	**<0.0001**
Anemia	5181 (2.40%)	3971 (2.14%)	1210 (4.05%)	**<0.0001**
Rheumatoid arthritis/collagen vascular diseases	5031 (2.33%)	3903 (2.10%)	1128 (3.77%)	**<0.0001**
Congestive heart failure	1996 (0.93%)	1611 (0.87%)	385 (1.29%)	**<0.0001**
Chronic pulmonary disease	32107 (14.89%)	25144 (13.53%)	6963 (23.30%)	**<0.0001**
Coagulopathy	1070 (0.50%)	863 (0.46%)	207 (0.69%)	**<0.0001**
Diabetes	31734 (14.71%)	26579 (14.31%)	5155 (17.25%)	**<0.0001**
Opioid dependence/abuse	464 (0.22%)	309 (0.17%)	155 (0.52%)	**<0.0001**
Other Drug dependence /abuse	1106 (0.51%)	776 (0.42%)	330 (1.10%)	**<0.0001**
Hypertension	89823 (41.65%)	75413 (40.59%)	14410 (48.21%)	**<0.0001**
Hypothyroidism	17791 (8.25%)	13753 (7.40%)	4038 (13.51%)	**<0.0001**
Liver disease	1748 (0.81%)	1361 (0.73%)	387 (1.29%)	**<0.0001**
Fluid/electrolyte disorders	3708 (1.72%)	2920 (1.57%)	788 (2.64%)	**<0.0001**
Obstructive sleep apnea	15674 (7.27%)	12038 (6.48%)	3636 (12.17%)	**<0.0001**
Other neurological disorders	6581 (3.05%)	4774 (2.57%)	1807 (6.05%)	**<0.0001**
Obesity	21015 (9.74%)	16597 (8.93%)	4418 (14.78%)	**<0.0001**
Peripheral vascular disorders	2371 (1.10%)	1989 (1.07%)	382 (1.28%)	**0.0014**
Pulmonary circulation disorders	433 (0.20%)	339 (0.18%)	94 (0.31%)	**<0.0001**
Renal failure	2370 (1.10%)	1948 (1.05%)	422 (1.41%)	**<0.0001**
Valvular disease	4354 (2.02%)	3593 (1.93%)	761 (2.55%)	**<0.0001**
Weight loss	344 (0.16%)	270 (0.15%)	74 (0.25%)	**<0.0001**
Osteoporosis	4250 (1.97%)	3243 (1.75%)	1007 (3.37%)	**<0.0001**
Tobacco use	66197 (30.69%)	54608 (29.39%)	11589 (38.77%)	**<0.0001**
Anxiety	14474 (6.71%)	9145 (4.92%)	5329 (17.83%)	**<0.0001**
**Number of comorbidities**				**<0.0001**
0	56271 (26.09%)	52721 (28.38%)	3550 (11.88%)	
1	64451 (29.88%)	56866 (30.61%)	7585 (25.38%)	
2	48730 (22.59%)	40857 (21.99%)	7873 (26.34%)	
3+	46232 (21.44%)	35351 (19.03%)	10881 (36.40%)	
**Hospital characteristics**				
**Hospital bedsize**				**<0.0001**
Large (>450) (ref)	132522 (61.76%)	114422 (61.89%)	18100 (60.93%)	
Medium (250–450)	48372 (22.54%)	41697 (22.55%)	6675 (22.47%)	
Small (<250)	33686 (15.70%)	28757 (15.55%)	4929 (16.59%)	
**Location/teaching status**				**<0.0001**
Rural (ref)	10314 (4.81%)	8863 (4.79%)	1451 (4.88%)	
Urban nonteaching	95723 (44.61%)	83026 (44.91%)	12697 (42.75%)	
Urban teaching	108543 (50.58%)	92987 (50.30%)	15556 (52.37%)	
**Hospital region**				**<0.0001**
Northeast (ref)	32163 (14.91%)	27580 (14.84%)	4583 (15.33%)	
Midwest	48478 (22.48%)	40611 (21.86%)	7867 (26.32%)	
South	98112 (45.49%)	85355 (45.94%)	12757 (42.68%)	
West	36931 (17.12%)	32249 (17.36%)	4682 (15.66%)	
**Hospital volume (surgeries/year)**				**0.0002**
Low (ref) (<153)	160916 (74.61%)	138355 (74.47%)	22561 (75.48%)	
High (> = 153)	54768 (25.39%)	47440 (25.53%)	7328 (24.52%)	

Significant value was in bold.

ACDF, anterior cervical discectomy and fusion.

Table 2 shows patients’ postoperative outcomes based on depression status. Significant differences were found between depressive and non-depressive patients in unfavorable discharge, prolonged LOS, complications (i.e., any complication, postoperative acute or chronic pain, delirium, dysphagia, cardiovascular complications, venous thromboembolic, respiratory complications and pneumonia, bleeding complications, infection/sepsis and hospital costs), with higher proportions observed in depressive patients (all P < 0.01) (Table 2).

**Table 2:** Post-surgical outcomes of the population by depression status.

	Total (n = 215684)	Depression		P-value
		No (n = 185795)	Yes (n = 29889)	
**Unfavorable Discharge**				**<0.0001**
N (Home/ routine / short-term hospital)	211156 (97.90%)	182082 (98.00%)	29074 (97.27%)	
Y (Long-term facility)	4415 (2.05%)	3618 (1.95%)	797 (2.67%)	
Die in hospital	113 (0.05%)	95 (0.05%)	18 (0.06%)	
**Prolonged LOS (excluded die in hospital)**				**<0.0001**
N (0–2 day)	192841 (89.41%)	166896 (89.83%)	25945 (86.80%)	
Y (3+ days)	22843 (10.59%)	18899 (10.17%)	3944 (13.20%)	
**Complication**				
Any	21663 (10.04%)	17438 (9.39%)	4225 (14.14%)	**<0.0001**
Postoperative acute or chronic pain	1396 (0.65%)	1081 (0.58%)	315 (1.05%)	**<0.0001**
Delirium	336 (0.16%)	264 (0.14%)	72 (0.24%)	**<0.0001**
Dysphagia	6583 (3.05%)	5496 (2.96%)	1087 (3.64)	**<0.0001**
Cardiovascular complications	2953 (1.37%)	2436 (1.31%)	517 (1.73%)	**<0.0001**
Venous thromboembolic	254 (0.12%)	203 (0.11%)	51 (0.17%)	**0.0041**
Respiratory complication and pneumonia	3731 (1.73%)	3002 (1.62%)	729 (2.44%)	**<0.0001**
Acute kidney injury	553 (0.26%)	466 (0.25%)	87 (0.29%)	0.2014
Digestive System	273 (0.13%)	221 (0.12%)	52 (0.17%)	**0.0130**
Bleeding complication	6996 (3.24%)	5516 (2.97%)	1480 (4.95%)	**<0.0001**
Infectious/sepsis	3604 (1.67%)	2778 (1.50%)	826 (2.76%)	**<0.0001**
**Hospital cost**	46971.3±31239.2	46719.7±30866.6	48535.4±33420.7	**<0.0001**

Significant value was in bold.

### Associations between surgical outcomes and clinical depression

Table 3 summarizes results of univariate and multivariate regression analysis on associations between depression and postoperative outcomes. After adjusting for confounding factors, depression was significantly associated with higher odds of unfavorable discharge (aOR: 1.311, 95% CI: 1.193–1.439), prolonged LOS (aOR: 1.152, 95% CI: 1.101–1.205), any complication (aOR: 1.232, 95% CI: 1.174–1.292), postoperative acute or chronic pain (aOR: 1.432, 95% CI: 1.239–1.656), dysphagia (aOR: 1.105, 95% CI: 1.022–1.193), respiratory complications and pneumonia (aOR: 1.153, 95% CI: 1.045–1.273), bleeding complications (aOR: 1.085, 95% CI: 1.083–1.087), infection/sepsis (aOR: 1.529, 95% CI: 1.390–1.682), and increased hospital costs (beta: 1080.640, 95% CI: 645.552–1515.727) than non-depression (Table 3**)**.

**Table 3:** Univariate and multivariate analysis of the associations between outcomes and depression.

Outcome	Depression	
	OR/beta (95% CI)	aOR/beta (95% CI)
**Unfavorable discharge**	**1.375 (1.273–1.484)**	**1.311 (1.193–1.439)**
**Prolonged LOS**	**1.344 (1.295–1.394)**	**1.152 (1.101–1.205)**
**Complication**	**1.589 (1.533–1.648)**	**1.232 (1.174–1292)**
Postoperative acute or chronic pain	**1.820 (1.604–2.065)**	**1.432 (1.239–1.656)**
Delirium	**1.697 (1.307–2.203)**	1.241 (0.900–1.712)
Dysphagia	**1.238 (1.159–1.323)**	**1.105 (1.022–1.193)**
Cardiovascular complications	**1.325 (1.205–1.459)**	1.064 (0.951–1.191)
Venous thromboembolic	**1.563 (1.149–2.125)**	1.355 (0.924–1.986)
Respiratory complication and pneumonia	**1.522 (1.403–1.652)**	**1.153 (1.045–1.273)**
Acute kidney injury	1.161 (0.923–1.460)	0.792 (0.590–1.063)
Digestive System	**1.463 (1.082–1.980)**	1.185 (0.829–1.694)
Bleeding complication	**1.703 (1.606–1.806)**	**1.085 (1.083–1.087)**
Infectious/sepsis	**1.872 (1.731–2.026)**	**1.529 (1.390–1.682)**
**Hospital cost**	**1815.760 (1434.25–2197.26)**	**1080.640 (645.552–1515.727)**

**Unfavorable discharge:** Adjusting for age, income, race, insurance status / primary payer, diagnosis, all comorbidities, hospital bedsize, location/teaching status, hospital region, and hospital volume.

**Prolonged LOS:** Adjusting for age, gender, income, race, insurance status / primary payer, diagnosis, procedure, all comorbidities, hospital bedsize, location/teaching status, hospital region, and hospital volume.

**Any complication:** Adjusting for age, gender, income, race, insurance status / primary payer, diagnosis, procedure, all comorbidities, hospital bedsize, location/teaching status, hospital region, and hospital volume.

**Postoperative acute or chronic pain:** Adjusting for age, gender, income, race, insurance status / primary payer, diagnosis, procedure, all comorbidities, hospital bedsize, location/teaching status, hospital region, and hospital volume.

**Delirium:** Adjusting for age, gender, insurance status / primary payer, procedure, and all comorbidities.

**Dysphagia:** Adjusting for age, gender, income, race, insurance status / primary payer, diagnosis, procedure, all comorbidities, hospital bedsize, location/teaching status, hospital region, and hospital volume.

**Cardiovascular complications:** Adjusting for age, gender, race, insurance status / primary payer, diagnosis, procedure, and all comorbidities.

**Venous thromboembolic:** Adjusting for age, gender, race, insurance status / primary payer, all comorbidities, hospital bedsize, hospital region, and hospital volume.

**Respiratory complication and pneumonia:** Adjusting for age, gender, race, insurance status / primary payer, and all comorbidities.

**Acute kidney injury:** Adjusting for age, gender, income, race, insurance status / primary payer, diagnosis, all comorbidities, hospital bedsize, hospital region, and hospital volume.

**Digestive System:** Adjusting for age, race, insurance status / primary payer, diagnosis, and all comorbidities.

**Bleeding complication:** Adjusting for age, gender, income, race, insurance status / primary payer, procedure, all comorbidities, hospital bedsize, and hospital region.

**Infectious/sepsis:** Adjusting for age, gender, income, race, insurance status / primary payer, procedure, all comorbidities hospital bedsize, location/teaching status, hospital region, and hospital volume.

**Hospital cost:** Adjusting for age, gender, income, race/ethnicity, insurance status / primary payer, diagnosis, procedure, comorbidities, hospital bedsize, location/teaching status, hospital region and hospital volume.

### Associations between surgical outcomes and clinical depression by procedure type

Stratified analyses on the associations between depression and postoperative outcomes by procedure type are shown in Table 4. Patients were stratified into two subgroups: primary and revision surgery. For patients receiving primary surgery, after adjusting for confounders, depression was significantly associated with prolonged LOS (aOR: 1.150, 95% CI: 1.098–1.203), increased likelihood of any complication (aOR:1.233, 95% CI: 1.174–1.294) and postoperative acute and chronic pain (aOR:1.927,95% CI: 1.692–2.194). For patients undergoing revision surgery, however, no significant associations were found for prolonged LOS, presence of any complication or postoperative pain (Table 4).

**Table 4:** Associations between postoperative outcomes and depression, stratified by procedure type.

Subgroup	Prolonged LOS		Any complication		Postoperative acute and chronic pain	
	OR (95% CI)	aOR (95% CI)	OR (95% CI)	aOR (95% CI)	OR (95% CI)	aOR (95% CI)
Primary	**1.338 (1.290–1.389)**	**1.150 (1.098–1.203)**	**1.586 (1.529–1.645)**	**1.233 (1.174–1.294)**	**2.011 (1.810–2.235)**	**1.927 (1.692–2.194)**
Revision	**1.396 (1.100–1.772)**	1.218 (0.908–1.633)	**1.521 (1.199–1.931)**	1.158 (0.843–1.590)	1.861 (0.810–4.275)	1.107 (0.350–3.500)

**Prolonged LOS:** Adjusting for age, gender, income, race, insurance status / primary payer, diagnosis, all comorbidities, hospital bedsize, location/teaching status, hospital region, and hospital volume.

**Any complication:** Adjusting for age, gender, income, race, insurance status / primary payer, diagnosis, all comorbidities, hospital bedsize, location/teaching status, hospital region, and hospital volume.

**Postoperative acute or chronic pain:** Adjusting for age, gender, income, race, insurance status / primary payer, diagnosis, all comorbidities, hospital bedsize, location/teaching status, hospital region, and hospital volume.

## Discussion

In the present assessment of the clinical characteristics and in-hospital outcomes of hospitalized patients with and without depression who underwent ACDF for degenerative cervical spine disease, the prevalence of depression nearly doubled between 2005 and 2014 in NIS population of the US. Pre-surgical clinical depression was independently associated with increased risk of post-surgical acute or chronic pain. However, depression was only associated with a slight risk increase in unfavorable discharge, prolonged LOS, any complication and hospital costs than in those without depression. More specifically, depression significantly but not strongly increased the risk for respiratory complications and pneumonia, dysphagia, bleeding complications and infection/sepsis after adjustment for confounders. No associations were found between pre-surgical clinical depression and postoperative delirium, cardiovascular and venous thromboembolic complications. In the subgroup analysis of patients receiving revision surgery, the associations between depression, post-surgical pain, LOS and any complication lost their significance.

Only a limited number of studies have evaluated in-patient outcomes after degenerative cervical spine surgery associated with depression, and no studies to date have reported associations in all outcomes assessed in the present study (i.e., acute or chronic pain, unfavorable discharge, prolonged hospital stay, postoperative complications, hospital costs). In a single-center study, among 264 patients undergoing ACDF for degenerative cervical pathology, presurgical depressive symptoms were associated with more pain and disability pre- and postoperatively than in non-depressed patients, but degrees of improvement were similar in both groups [21]. Mayo et al. [16] failed to show any association between preoperative mental health score assessment and improvement in self-reported outcomes as determined by the Neck Disability Index (NDI) or Visual Analogue Scale Neck (VAS Neck) after ACDF among 52 patients. A retrospective cohort study analyzing 106 patients undergoing ACDF found that patients with greater degrees of depression before and after surgery demonstrated lower measures of quality of life (QOL) and less overall improvement in health status compared to those with less or no depression [13]. In a statewide study of patients receiving fusion for either cervical myelopathy or radiculopathy [12], about 25% had depression, anxiety, sleep issues and stress with higher rates of infection, readmission and/or revision surgery postoperatively. In contrast, patients with poorer baseline mental health who underwent ACDF exhibited similar improvement in clinical outcomes, return to work or satisfaction rates despite having greater pain and disability, compared to those with high preoperative mental health scores [15]. However, those studies were either of results from single institution, with different outcome measures from the present study, or depression was not analyzed separately from other important mental conditions such as anxiety, sleep problems or stress, which complicates the interpretation of their final results.

The most relevant previous study may be the one conducted by Harris et al. which investigated patients undergoing ACDF [22]. For mental health disorders, preoperative anxiety and depression were reported separately as in the present study. Harris et al. [22] reported that multiday hospitalization and non-home discharge were both higher in patients with underlying depression compared to those without, which essentially agrees with the present study despite a much smaller cohort size and some discordance between covariates controlled. Preoperative depression was also associated with higher readmissions and revision surgery, along with greater risk for chronic postoperative opioid consumption [22]. However, the inherent limitation due to lack of random sampling in the use of private database has limited the validity of generalizations and trend analysis in their study [23]. Nevertheless, results of the Harris study cited above and those of the present analysis together highlight the critical impact of depression in degenerative cervical spine surgery.

Meanwhile, we acknowledge that pain outcomes are often evaluated by analyzing the amounts of narcotics patients use postoperatively [22]. Outcomes of patients undergoing lumbar spine surgery, for example, demonstrate increased postoperative cumulative opioid use, emphasizing the link between preoperative depression and adverse surgical outcomes, while also pointing to the increased risk of chronic opioid use and decreased likelihood of ceasing opioid use [24]. In the present study, pre-surgical depression had the strongest impact on postoperative acute or chronic pain among all postoperative complications, and the relation was even stronger among patient received revision fusion, however, if viewed by claims codes, the status of postoperative opioid use could not be ascertained.

Levin et al. [25] presented a unique perspective in a cohort of 145 patients regarding the link between preoperative depression and poorer health-related QOL in their report of a negative association between preoperative depression and patients’ perceptions of doctor-patient communication, which deemed depression a risk factor for a poorer overall surgical and postoperative experience, especially as related to communication with their spine surgeons. We did not evaluate patients’ subjective satisfaction or QOL due to lack of information in the NIS database, though it is understandable that depressed patients may evaluate their total surgical experience differently from non-depressed given the overriding study results that recount poorer postoperative outcomes associated with depression.

Depressive disorders are described in the Malhi and Mann seminar [2] as recurrent lifelong illness in which incident episodes fluctuate during each patient’s lifetime, while recovery may lack stable progress increasing the challenges of detection, diagnosis and management. Patients with depressive disorders, whether or not they were previously diagnosed, constitute a special subgroup of those undergoing spinal surgeries, and may exhibit higher levels of back pain, postoperative complications and overall poorer functional outcomes; surgical outcomes are often improved, however, by identifying and treating these depressed patients prior to scheduling spinal surgery [26].

Although the US Preventative Services Task Force has consistently recommended psychological screening for patients undergoing spinal surgery, these guidelines are not followed by all surgeons [27]. One group of surgeons reported that pretreatment of depression prior to cervical spine surgery contributed to improving patients’ perceptions of their postoperative health status [28]. As the prevalence of comorbid depression increased in patients undergoing degenerative cervical spine surgery, as shown in NIS data analyzed in the present study, we believe that the results suggest giving more attention to preoperative depression in this population.

### Strengths and limitations

Results of the present study are strengthened by the use of the nationally representative and continuous database, which allows outcomes to be assessed in the largest cohort amongst the medical literature and results to be generalized to the US population. Another strength is the focus on a relatively homogenous patient group and conducting stratified analyses for primary and revision procedure, which provides more applicable results than those of prior reports. Nevertheless, certain limitations are noted, including the use of retrospective analysis, which may limit the inference of causality. The NIS data lacks outpatient follow-up. The use of administrative claims data also means that detailed information such as the phenotypes or duration of depression of individual patients could not be identified and therefore could not be controlled for. Also, it could not be determined whether patients who were diagnostically coded as depression actually were undergoing treatment or receiving antidepressant medications at the time of surgery. The initial severity of cervical myelopathy and radiculopathy and of individual comorbidities cannot be acquired adequately using the coding system. The data also lack information on intraoperative variables such as operative time or laboratory parameters, which prevents more detailed analyses. Confirmation of the results of the present study requires further prospective multicenter studies in geographically diverse populations undergoing degenerative cervical spine surgery.

### Conclusions

Pre-surgical clinical depression in patients who have undergone primary ACDF for degenerative cervical spine disease predicts post-surgical acute or chronic pain, a slightly prolonged LOS and presence of any complication. Results of this study accentuate associations between depression and post-ACDF outcomes, which may help to improve treatment plans, healthcare delivery and policies surrounding the care of patients receiving cervical spine surgery. These results also may be useful in developing strategies to identify and treat depressive symptoms preoperatively, looking toward improving post-surgical outcomes.

## Supporting information

S1 Table ICD-9 and CCS codes used in cohort definitions and identification of postoperative complications. (DOCX)
