# Supplementary material for: A national snapshot of the impact of clinical depression on post-surgical pain and adverse outcomes after anterior cervical discectomy and fusion for cervical myelopathy and radiculopathy: 10-year results from the US Nationwide Inpatient Sample
Source: PLoS One. 2021 Oct 15;16(10):e0258517. doi: 10.1371/journal.pone.0258517 (PMC8519476; doi:10.1371/journal.pone.0258517)
Supplement: S1 Table — (DOCX) [file pone.0258517.s001.docx]

| **S1 Table.** ICD-9 and CCS codes used in cohort definitions and identification of postoperative complications. | | |
| --- | --- | --- |
| Variable |  | Codes |
| **Inclusions** | | |
| Cervical myelopathy and radiculopathy  ACDF |  | 721.0, 721.1, 722.0, 722.4, 722.71, 722.81, 722.91, 723.x  81.02, 81.32 |
| PCDF | | 81.03, 81.33 |
|  |  |  |
| Depression |  | 296.2, 296.3, 296.90, 300.4, 309.0, 309.28, 311 |
| **Exclusions** | | |
| Metastatic or nonmetastatic cancer, lymphoma, or leukemia | | 140.x-172.x, 174.x-195.8, 196.x-199, 200.x-208.x |
| Schizophrenia | | 295 |
| Bipolar disorder | | 296.0, 296.4-296.6, 296.7, 296.8, 301.13 |
| Vertebral fracture | | 805.00-806.9 |
| Trauma | | 839.0-839.5, 952.0-952.9 |
| Paralysis | | 334.1, 342.0-344.9, 438.2-438.5, 780.72 |
| Multi-level fusion | | 81.63, 81.64 |
| **Comorbidities** | | |
| Opioid dependence/abuse | | 338.18, 338.28 |
| Other Drug dependence /abuse  Obstructive sleep apnea  Osteoporosis  Tobacco use  Anxiety | | 304.1-304.6, 304.8-304.9, 305.2-305.4, 305.6-305.9  327.23, 780.57  733.0  305.1, V15.82  300.00-300.02, 300.09, 308.0-308.9, 309.81-309.83, 309.89 |
| **Postoperative complications** | | |
| Postoperative acute or chronic pain | | 338.18, 338.28 |
| Delirium | | 292.81, 293.0, 293.1, 293.8, 293.9, 780.97 |
| Dysphagia | | 787.2 |
| Cardiovascular complications | | 248.xx, 410.xx, 427.5, 785.xx, 997.1, 997.02, 997.09, 998.0, 100CCS |
| Venous thromboembolic complications | | 415.x, 451.11, 451.19, 451.2, 451.81-84, 451.89, 451.9, 453.x, 997.2 |
| Respiratory complications and pneumonia | | 514.x, 518.xx, or 512.x, 997.3, 997.31, 997.32, 997.39, 122CCS |
| Acute kidney injury | | 584.5-584.9, 157CCS, V45.1 |
| Digestive system complications | | 578.x, 560.1, 008.45 |
| Bleeding complications | | 285.x, 998.1, 998.2, 94.04 |
| Infection / Sepsis | | 038.x, 041.x, 320.x, 324.1, 481-486, 507.0, 595.0, 790.7, 995.9, 996.64, 997.31, 998.59, 999.31 |

ACDF, Anterior cervical discectomy and fusion; CCS, Clinical Classifications Software codes; PCDF, Posterior cervical discectomy fusion.
